# *In Vitro* Fertilization, Levels of Pro-Inflammatory Factors and Lipid Peroxidation

**DOI:** 10.22074/ijfs.2015.4541

**Published:** 2015-10-31

**Authors:** Ferdag Yildizfer, Orkide Donma, Mustafa Yen, Ozlem Ekmekci, Zubeyde Asli Karatas Kul, Zafer Keser, Ahmet Esat Imal, Emin Cagil, Murat Mengi, Hakan Ekmekci, Sezai Sahmay, Burcin Nalbantoglu, Mustafa Metin Donma

**Affiliations:** 1 Cerrahpasa Medical Faculty, Istanbul University, Istanbul, Turkey; 2 Ministry of Health, Diyarbakir Education and Research Hospital, Diyarbakir, Turkey; 3 University of Texas, Health Science Center at Houston, Houston, USA; 4 Tufts Medical Center, Molecular Oncology Research Institute, Boston, USA; 5 Faculty of Medicine, Namik Kemal University, Tekirdag, Turkey

**Keywords:** Infertility, Leptin, Resistin, TNF-α, Lipid Peroxidation

## Abstract

**Background:**

Infertility is a problem concerning 10-15% of the individuals in the fertile period. This study investigated effects of proinflammatory factors as well as lipid hydroperoxides (LPO) levels upon *in vitro* fertilization (IVF) success.

**Materials and Methods:**

In this prospective, non-randomized, controlled clinical study, sera obtained from 26 fertile (group-1), 26 infertile women before (group-2) and after (group-3) IVF treatment were analyzed. Leptin, leptin receptor, resistin, tumor necrosis factor-alpha (TNF-α), and C-reactive protein (CRP) were analyzed using enzyme-linked immunosorbent assay (ELISA). LPO was determined spectrophotometrically. Mann- Whitney U test, paired samples t test, Wilcoxon signed-rank test as well as Pearson correlation analysis by SPSS were performed for statistical analysis.

**Results:**

TNF-α, resistin and LPO levels increased (P=0.020, P=0.003, P=0.001, respectively) in group-3 compared to group-2. A significant increase in LPO was noted both in group-2 and -3 compared to controls (P=0.000). LPO were higher in non-pregnants than pregnants in group-2. For pregnants, significant correlations were observed between leptin and resistin in group-2 and TNF-α and leptin in group-3. None of these correlations were found for the women, who could not conceive.

**Conclusion:**

LPO, leptin-resistin correlation, associations with TNF-α may be helpful during the interpretation of IVF success rates.

## Introduction

Infertility can be defined as inability to conceive despite sexual relationship without contraception for 1 year. This problem concerns about 10-15% of the individuals in the fertile age (1).

To have a family and a child is well-accepted and a desired status all over the world. They are important for the development of the community and continuity of the generation. Couples with an enthusiasm of having a child but facing the problem of infertility, experience a decrease in communication, belief of health, social esteem and self-confidence, and become disappointed about expectations for the future. After the diagnosis of infertility is made, people ignore all areas of their lives and concentrate on the matter and the methods used in the processes of diagnosis and treatment, and particularly for the women, it becomes a painful chain of physical and emotional events (2).

*In vitro* fertilization (IVF) can be defined as one of the assisted reproductive techniques medically applied on oocyte, sperm or embryo cells *in vitro* in order to develop pregnancy (3, 4). Cytokines as key modulators of the immune system appear to modulate other regulatory systems. They also contribute to regulation of the ovarian cycle (5). A proinflammatory cytokine tumor necrosis factoralpha (TNF-α) and C-reactive protein (CRP), one of the acute phase reactants, can increase resistin expression (6-9). CRP is a negative regulator of functions of human leptin (10). Resistin is synthesized mostly by inflammatory cells such as macrophages and correlated with TNF-α (9). Resistin levels are capable of increasing expression of TNF-α via nuclear factor (NF)-κβ-dependent pathway (6, 8, 11-13). Leptin, a proinflammatory factor, regulates food intake and energy expenditure (14). It is also linked to reproductive functions (15). Leptin levels may be used as predictive markers of assisted reproductive technology (ART) (16, 17). It has been demonstrated that combined exposure of human monunuclear cells to high concentrations of insulin and leptin for 24 hours *in vitro* stimulates resistin and TNF-α protein expression (12). Leptin level is elevated in cases associated with high levels of TNF-α that increases serum leptin concentrations (14, 18).

Studies on reactive oxygen molecules (ROM) during the course of this process and their relationship with proinflammatory factors have gained importance in recent years (19, 20). Shift of the equilibrium between pro-oxidants and anti-oxidants towards pro-oxidants results in oxidative stress. Effects of oxidative stress on the stages of reproduction like oocyte maturation and follicular development are important from the IVF success point of view (21). Its importance is emphasized by the conditions providing low oxygen during IVF application. Since the role of oxidative stress on infertility has not been fully cleared yet, effects of lipid peroxidation upon various stages of IVF process and the contribution of some cytokines and hormones upon the process are noteworthy.

The aim of this study was to investigate the profiles of some pro-inflammatory factors, cytokines and hormones, such as CRP, TNF-α, leptin as well as resistin, known to be involved in the process of inflammation, to evaluate their relationship with lipid hydroperoxides, the markers of early lipid peroxidation and to assess their associations with female infertility.

## Materials and Methods

In this prospective, non-randomized, controlled clinical study, the blood samples from 70 women, who consulted to the IVF Center, Obstetrics and Gynaecology Department, Cerrahpasa Medical Faculty, University of Istanbul, Istanbul, Turkey, with the complaint of infertility were used. They also had the features of being between the ages of 23 and 40, being married for 3 years, having social security for 5 years and having two times of intrauterine insemination before, while they were taken for analysis prior to the beginning of the treatment in order to determine the suitability for the participation into the study.

Patient history and gynaecological exam, routine biochemical tests, ultrasonography, serology, basic infertility tests [spermiogram, hormonal tests and hysterosalpingography (HSG)] were performed to evaluate the causes of infertility before the treatment in order to enlighten the source of the problem.

Causes of reduced female fertility included decreased ovarian reserve, anovulation, uterine disorders other than endometriosis, fertility-sparing surgery with unilateral salpingo-oophorectomy, methylene tetrahydrofolate reductase gene mutation, unexplained reasons and presence of more than one factor. Patients with polycystic ovary syndrome (PCOS) were also included in the study population, with the result that both pregnant and non-pregnant groups had a proportionate distribution to eliminate the effects of their possible contribution in terms of inflammation.

A signed written informed consent was obtained from all participants prior to the study. Procedures were carried out in accordance with Declaration of Helsinki. This project was approved by the Ethics Committee and Institutional Board of Cerrahpasa Medical Faculty, Istanbul University, Istanbul, Turkey.

Twenty-six women, who had given a birth without any medication constituted the control group (group 1). The blood samples from 70 infertile women were collected during the early follicular phase (the 3rd day of the cycle) before the onset of the intervention. A total of 26 individuals managed to complete the process with the appropriate respond to treatment. Their "pre-IVF samples" (group 2) out of 70 were included into the study to constitute the paired data with the "post-IVF samples" (group 3) taken on the 15^th^ day of the application of embryo transfer from these 26 women. There was no statistically significant difference between the age and body mass index (BMI) values of the control and patient groups (P=0.909, P=0.431, respectively).

Anthropometric measurements and demographic characteristics of the women participated in the study were recorded. Blood samples were taken into sterile vacuum operated tubes at 08:00 – 10:00 am while fasting before the IVF treatment on the 3rd day of the menstruation (follicular phase) and on the 15^th^ day after the embryo transfer, and were centrifuged in 2000 rpm for 10 minutes. Serum samples were stored at -80˚C until assayed.

Serum anti-mullerian hormone (AMH), inhibin B, follicle stimulating hormone (FSH), luteinizing hormone (LH), estradiol (E_2_), prolactin, and thyroid stimulating hormone (TSH) levels were determined in all women. The levels of TNF-α (Human TNF-α Elisa Kit, Assaypro, USA), resistin (Human Resistin Elisa Kit, Assaypro USA), leptin (Human Leptin Elisa Kit, Assaypro, USA), leptin receptor (Human Leptin Receptor Elisa Kit, BioVendor, EU), and high sensitive -C reactive protein (hs-CRP) (CRP HS ELISA Kit, DRG Int, Inc. USA) were determined by commercially available enzyme-linked immunosorbent assay (ELISA) kits. Lipid hydroperoxide levels, one of the important markers of oxidative stress, were determined by a spectrophotometric method [Lipid Hydroperoxide (LPO) Assay Kit, Cayman Chem Comp., USA].

All samples were assayed using the AssayMax Human TNF-alpha ELISA kit (AssayPro, USA). The intra-assay and inter-assay coefficients of variation (CV) were 5.5 and 7.0%, respectively, using the AssayMax Human Resistin ELISA kit (Assaypro, USA). The intra-assay and inter-assay CV were 4.0 and 7.2%, respectively, using the AssayMax Human Leptin ELISA kit (Assaypro, USA). The intra-assay and inter-assay CV were 4.0 and 7.7%, respectively, using the Human Leptin Receptor ELISA kit (BioVendor Research and Diagnostic Products, EU). The intra-assay and inter-assay CV were 7.2 and 9.8%, respectively, using the hs-CRP ELISA kit (DRG International, Inc., USA). The intra-assay and inter-assay CV were 4.1 and 7.5%, respectively, using the Lipid Hydroperoxide (LPO) Assay kit (Cayman Chemical Company, USA).

### Statistical analyses

Statistical analyses were performed using the Statistical Package for Social Sciences (SPSS, SPSS Inc., Chicago, IL, USA) software package. Data were analyzed using descriptive-analytic tests. Parametric variables were represented as mean and standard error (SE), and categorical data were represented by number (n) and percentage (%). The values for arithmetical mean, standard deviation (SD) and SE were calculated for the pregnant and non-pregnant women in pre-IVF and post-IVF groups as well as for the participants in control group. Mann-Whitney U test, paired sample t test, Wilcoxon signed-rank test as well as Pearson correlation analysis were performed. P values less than 0.05 were considered significantly.

## Results

IVF-applied group had a mean age (mean ± SE) of 31.2 ± 1.4 years and BMI of 25.4 ± 2.8 kg/m^2^. Control group was consisted of healthy women who took no medication, had no illnesses, were spontaneously-conceived and volunteered. Mean age of this group was 31.4 ± 1.5 years and BMI value was 24.1 ± 1.1 kg/m^2^. There was no statistically significant difference between the ages and BMI values of the groups.

Eight of 26 women conceived after application of IVF treatment (30.8%). Two of them were concluded with medical abortion due to unembryonic/ empty sac pregnancy. Six of 26 women finished the period with live birth (23.1%).

In table 1, some demographical and clinical parameters in non-pregnant and pregnant women are summarized.

Values for mean ± SE for the parameters of control, pre- and post-IVF groups as well as P values that define the statistical differences between the groups are shown in table 2.

The values of LPO (P=0.000) and leptin receptor (P=0.01) between control and pre-IVF groups showed statistically significant differences.

There were statistically significant differences between LPO (P=0.000), CRP (P=0.023) and resistin (P=0.002) levels obtained in control and post-IVF groups.

The differences between pre-IVF and post-IVF levels of resistin, LPO (P=0.003, P=0.001, respectively) and TNF-α (P=0.020) were statistically significant.

As far as the values for the parameters in the pregnancy (+) and pregnancy (-) groups of preand post- IVF women were considered, TNF-α showed a statistically significant increase after IVF in pregnancy (-) women (P=0.026). Similar post-IVF profiles were observed for the resistin (P=0.028, P=0.016, respectively) and LPO (P=0.046, P=0.003, respectively) levels in pregnancy (+) and pregnancy (-) groups. Table 3 shows the mean ± SE and p values of the nonpregnant and pregnant women in pre-IVF and post- IVF groups.

BMI had a positive correlation with leptin (r=0.615, P=0.001), a negative correlation with leptin receptor (r=-0.505, P=0.008) and a positive correlation with CRP (r=0.464, P=0.039). Also, leptin had a negative correlation with leptin receptor (r=-0.534, P=0.005) and a positive correlation with CRP (r=0.639, P=0.002) in the control group. In this group, resistin had also positive correlation with age (P≤0.05).

Before IVF, statistically significant correlations were observed between BMI and leptin (r=0.486, P=0.012) with resistin (r=0.517, P=0.007); between leptin and leptin receptor (r=-0.574, P=0.002) with resistin (P=0.047) and between TNF-α and LPO (P=0.016). After IVF, a significant correlation between BMI and leptin (r=0.641, P=0.000) was found. Strong correlations were detected for leptin (r=0.599, P=0.001) and LPO (r=0.715, P=0.000) between preand post-IVF values.

Important correlations were determined between BMI and leptin before (r=-0.547, P≤0.05) and after IVF (r=0.771, P≤0.01) for the women who were in the pregnancy (-) group. Before IVF, also, a statistically important relationship was observed between BMI and resistin (r=0.686, P≤0.01). An inverse association was noted between leptin and leptin receptor (r=-0.617, P≤0.01).

Women, who could conceive following IVF treatment showed significant correlations between leptin and resistin (r=0.874, P≤0.01) before IVF as well as leptin and TNF-α (r=0.841, P≤0.01) after IVF. None of these correlations were detected in the pregnancy (-) group.

**Table 1:** Comparison of some demographical and clinical parameters (mean ± SE) in non-pregnant and pregnant women


	Non-pregnant	Pregnant	P value

Age (Y)	31.3 ± 1.3	33.2 ± 1.5	≥ 0.05
Infertility period (Y)	7.4 ± 1.1	5.0 ± 1.2	≥ 0.05
BMI (kg/m^2^)	25.8 ± 1.7	24.9 ± 2.9	≥ 0.05
AMH (ng/ml)	4.2 ± 0.8	4.2 ± 1.0	≥ 0.05
LH (mIU/ml)	4.6 ± 1.1	3.3 ± 0.8	≥ 0.05
FSH (mIU/ml)	5.8 ± 0.3	4.9 ± 0.7	≥ 0.05
Prolactin (ng/ml)	16.7 ± 1.9	16 ± 3.2	≥ 0.05
Estradiol (pg/ml)	43.0 ± 4.0	37.5 ± 5.3	≥ 0.05
TSH (μIU/ml)	1.6 ± 0.2	1.8 ± 0.4	≥ 0.05
Inhibin-B (pg/ml)	100.2 ± 19.0	99.7 ± 3.5	≥ 0.05
Gonadotropin dose (IU)	2025 ± 190	1958 ± 346	≥ 0.05
Total oocytes (n)	8.6 ± 1.1	9.3 ± 1.9	≥ 0.05
Fertilized oocytes (n)	4.2 ± 0.7	6.0 ± 0.9	≥ 0.05
Transferred embryos (n)	2.0 ± 0.2	2.2 ± 0.3	≥ 0.05


SE; Standard error, BMI; Body mass index, AMH; Anti-mullerian hormone, LH; Luteinizing hormone, FSH; Follicule stimulating hormone and TSH; Thyroid stimulating hormone.

**Table 2:** The mean ± SE and P values of the control and IVF groups


Parameter		Control (Group 1)	Pre-IVF (Group 2)	Post-IVF (Group 3)	P value

TNF-α (pg/ml)	Mean ± SE	9.7 ± 0.8	7.2 ± 0.5	9.8 ± 0.9	≤ 0.05 G2 vs. G3 ≤ 0.05 G1 vs.G2
Leptin (ng/ml)	Mean ± SE	42.0 ± 6.4	62.6 ± 10.5	52.7 ± 6.8	≥ 0.05
Leptin receptor (ng/ml)	Mean ± SE	42.3 ± 3.0	32.0 ± 2.4	42.7 ± 5.7	≤ 0.05 G1vs.G2
CRP (mg/L)	Mean ± SE	3.8 ± 0.5	3.4 ± 0.4	7.0 ± 0.8	≤ 0.05 G1vs.G3
Resistin (ng/ml)	Mean ± SE	12.2 ± 1.1	12.5 ± 1.5	22.2 ± 2.9	≤ 0.01 G1vs.G3 ≤ 0.01 G2 vs. G3
LPO (nmol)	Mean ± SE	1.5 ± 0.3	4.4 ± 0.3	5.3 ± 0.2	≤ 0.001 G1vs.G2 ≤ 0.001 G1vs.G3 ≤ 0.01 G2 vs. G3


SE; Standard error, IVF; *In vitro* fertilization, TNF-α; Tumor necrosis factor alpha, CRP; C-reactive protein and LPO; Lipid hydroperoxides.

**Table 3:** The mean ± SE and P values of the non-pregnant and pregnant women in pre-IVF and post-IVF groups


Parameter		Non-pregnant			Pregnant		
		Pre-IVF	Post-IVF	P value	Pre-IVF	Post-IVF	P value

TNF-α (pg/ml)	Mean ± SE	7.1 ± 0.5	10.1 ± 0.9	≤ 0.05	7.8 ± 0.5	8.4 ± 0.7	≥ 0.05
Leptin (ng/ml)	Mean ± SE	64.9 ± 10.5	51.2 ± 6.7	≥ 0.05	54.6 ± 6.9	57.3 ± 7.5	≥ 0.05
Leptin receptor (ng/ml)	Mean ± SE	31.6 ± 2.5	43.2 ± 5.5	≥ 0.05	33.1 ± 2.7	40.8 ± 5.1	≤ 0.05
CRP (mg/L)	Mean ± SE	3.8 ± 0.5	8.4 ± 1.0	≥ 0.05	2.8 ± 0.4	7.2 ± 0.8	≥ 0.05
Resistin (ng/ml)	Mean ± SE	12.9 ± 1.7	20.6 ± 2.6	≤ 0.05	11.2 ± 1.1	27.8 ± 3.4	≤ 0.05
LPO (nmol)	Mean ± SE	4.5 ± 0.3	5.3 ± 0.3	≤ 0.01	4.1 ± 0.2	5.3 ± 0.3	≤ 0.05


SE; Standard error, IVF; *In vitro* fertilization, TNF-α; Tumor necrosis factor alpha, CRP; C-reactive protein and LPO; Lipid hydroperoxides.

## Discussion

Infertile couples have to face emotional and economical sides of the problem, because success rates of IVF trials have not reached to the desired level, yet. Immunologic factors may contribute to unexplained losses and thus, studies on the matter are being accelerated.

Cytokines are polypeptides that occur at the crossroads of immunological pathways. Maternal inflammatory response plays an important role in the early stages of pregnancy; however, there is no consensus on the roles of inflammatory parameters within this period (22).

In this study, mean TNF-α levels were found higher (P≤0.05) in the pregnancy (-) group than pregnancy (+) group after IVF. This situation reminds us a question whether the success rates of IVF applications in infertile women can be increased by use of TNF-α blockers.

It was reported that use of TNF-α inhibitors and intravenous immunoglobulins (IVIG) in young infertile women improved the result of IVF application and increased the success rates of IVF. TNF-α/ interleukin-10 (IL-10) elevation before pregnancy might relate with the risk of failure in IVF (23-25).

Serum resistin levels might be a good predictor of ovarian response in infertile women during IVF (7). In the present study, resistin levels were almost the same in the control and pre-IVF groups (12.2 ± 1.1 ng/ml vs. 12.5 ± 1.5 ng/ml). This level increased to 22.2 ± 2.9 ng/ml after IVF. This increase suggested that the profile of this parameter could be important.

Resistin shares several features with proinflammatory cytokines in humans and can partially contribute to regulation of inflammation and immunity. Macrophages incubated with recombinant resistin caused elevated production of TNF-α via the transcription factor NF-κβ dependent pathway (6, 13). Elevated TNF-α and resistin levels may contribute to increased inflammation, which may lead to poor quality oocytes and embryos (7, 26).

In the previous study, resistin was reported to increase the expression of TNF-α (27). In this study, post-IVF levels of TNF-α also increased in a parallel manner with resistin. CRP levels were similarly increased but much higher than the levels of TNF-α.

In a similar manner, CRP levels of post-IVF group were statistically higher than those of control and pre-IVF groups. In Pre-IVF group, a statistically significant difference was found between pregnancy (+) and pregnancy (-) groups (2.8 mg/L vs. 3.8 mg/L). It was noted that pre-IVF pregnancy (+) group had lower levels of CRP than the other group.

Probable effects between leptin and systemic inflammation are on-going discussion subject. Studies on culture cells and mouse models reported that human CRP prevented binding of leptin to its specific receptor and blocked the signal transduction. Thus, this parameter may weaken the physiologic function of leptin that contributes to "leptin resistance" (10, 28, 29).

Leptin, an adipocyte-derived hormone, does not only take role in the regulation of food intake, but is also involved in many reproductive functions including steroideogenic potential of ovary (15). Ovary is a target organ for leptin because leptin, its mRNA as well as its receptors are found in reproductive tissues (15, 30). Since leptin may influence follicular growth as well as oocyte development, leptin and leptin receptors were also investigated in this study.

In general terms, investigation of the effects of hormones that were applied in the extent of IVF treatment protocol showed decreases in leptin levels of the post-IVF group compared to pre-IVF values (52.7 ± 6.8 ng/ml vs. 62.6 ± 10.5 ng/ml). Pre-IVF levels of leptin decreased significantly in the pregnancy (+) group compared to the pregnancy (-) group (54.6 ng/ml vs. 64.9 ng/ml). Our results were consistent with the report stating that elevated leptin may exert adverse impacts on pregnancy success (15). Several investigations reported that high leptin is associated with low pregnancy rates in IVF cycles (16, 31). The effect of leptin on embryo quality is currently a controversial topic (30, 32, 33). However, it remains elucidated how elevated leptin concentrations negatively impact IVF outcome (31).

Certain studies (34-36) showed that soluble leptin receptor levels were inversely correlated with BMI. A similar relationship was found for the control group in this study. Levels of leptin receptor were higher in the post-IVF period (42.7 ± 5.7 ng/ml) compared with the pre-IVF period (32.0 ± 2.4 ng/ml), but there was no statistically significant difference between the levels in pre- IVF and post-IVF pregnancy (+) and pregnancy (-) groups.

In this study a positive correlation was found between levels of leptin and TNF-α in post-IVF pregnancy (+) women. This suggested that leptin may have a relationship with some other inflammatory parameters.

The best investigated adipocytokin up-tillnow is resistin. It is claimed that resistin takes role as an acute phase reactant due to its up-regulation in patients with severe sepsis and septic shock (10, 28, 37). However, there is little knowledge about its potential association with leptin. A finding that may contribute to this subject was obtained in this study. A strong pre-IVF relationship was determined between leptin and resistin in pregnancy (+) women following IVF application.

On the other hand, in a recently published article, leptin and resistin are reported as negative and positive outcome predictors, respectively, in women undergoing IVF (38). Our results were consistent with these findings. In pregnancy (+) group, as pre-IVF samples showed significant decreases in leptin concentrations, increased resistin levels were observed for post-IVF samples.

Oxidative stress may be used as a predictive marker in controlled ovarian stimulation success (39). Although it is not sufficient to measure LPO levels alone to interpret oxidative stress, they give some notion about the matter as the markers of early lipid peroxidation. Significant differences were found between LPO levels of the control group and pre-IVF as well as post- IVF groups. Also, a strong correlation was found between pre-IVF and post-IVF values of this parameter. Levels for this parameter were determined lower in pre-IVF pregnancy (+) group compared to pregnancy (-) group.

## Conclusion

In women, who ended up the IVF attempt with a successful pregnancy, a relationship between leptin and resistin is noted beside the association of these parameters with TNF-α. Relationships between leptin and resistin as well as TNF-α are expected, because double-sided effects are being observed among them. Resistin increases TNF-α, which in turn induces resistin. Leptin stimulates both resistin and TNF-α, that increases serum leptin concentrations. Association of TNF-α as well as resistin levels with quality of oocytes and embryos, and also influence of leptin upon follicular growth as well as oocyte development support the leptin-resistin and leptin-TNF-α correlations, which appear to be effective upon IVF outcome. Monitoring the levels of these parameters within the period, which follows IVF attempt may reveal more significant relationships.
